# Sap Flow and Environmental Responses Across *Salix psammophila* Stand Ages

**DOI:** 10.3390/plants15213317

**Published:** 2026-10-26

**Authors:** Min Han, Yuefeng Guo, Yong Gao, Wei Qi, Yang Song

**Affiliations:** 1 College of Desert Control Science and Engineering, Inner Mongolia Agricultural University, Hohhot 010018, China; 13314729077@163.com (M.H.); 13948815709@163.com (Y.G.); song.yang.01@foxmail.com (Y.S.); 2 State Key Laboratory of Water Engineering Ecology and Environment in Arid Area, Inner Mongolia Agricultural University, Hohhot 010018, China; 3 Inner Mongolia Autonomous Region Water Conservancy Development Center, Hohhot 010020, China; qiwemail@126.com

**Keywords:** Mu Us Sandy Land, sand willow, stand age, Sap flow rate (Q), environmental response

## Abstract

Water use and its environmental responses in artificially planted sand–fixing shrubs in arid and semi–arid regions are important for evaluating the sustainability of ecological restoration. This study investigated artificially planted *Salix psammophila* stands aged 3, 8, and 15 years in the Mu Us Sandy Land. From May to September 2025, branch sap flow rates were continuously monitored using the tissue heat balance method, while environmental factors, including solar radiation (Rs), air temperature (Ta), relative humidity (Rh), soil temperature (Ts), soil volumetric moisture content (Sh), wind speed (Ws), precipitation (Ra), and vapor pressure deficit (VPD), were simultaneously observed. Pearson correlation analysis, partial correlation analysis controlling for day of year (DOY), segmented regression, Random Forest (RF), and SHAP were used to compare the statistical associations and response patterns between sap flow and environmental factors among stands of different ages. The results showed that: (1) The daily mean branch sap flow rate during the growing season generally followed the order 8a > 3a > 15a, although the ranking varied among seasonal stages. Because the diameters of the monitored branches differed among stand ages, this result primarily reflects differences in branch–scale absolute sap flow rates among the sampled stands and cannot be directly equated with differences in hydraulic capacity per unit conducting tissue. (2) Sap flow in the 3a and 8a stands was strongly associated with Sh. In the 15a stand, sap flow was significantly negatively correlated with Ta, Ts, and VPD at the whole–growing–season scale; however, these relationships were no longer significant after controlling for DOY, indicating that they were largely influenced by common seasonal trends. (3) RF analysis showed that the relative predictive contributions of environmental variables differed among the stand–age–specific models. OOB–R^2^ ranged from 0.303 to 0.526, whereas CV–R^2^ values from 5–fold forward–chaining time–series cross–validation were below 0 for all models, indicating that the models could describe sap flow–environment relationships within the observation period to some extent but had limited temporal generalization to subsequent unseen periods. Overall, the sampled *S. psammophila* stands of different ages exhibited distinct sap flow–environment association patterns, with the 15a stand showing more pronounced seasonal dependence.

## 1. Introduction

In arid and semi–arid regions, water supply is limited while evapotranspiration demand is high, and vegetation water use therefore has important implications for regional water cycling, ecological restoration, and the stability of planted vegetation [1,2]. The Mu Us Sandy Land is located in a typical agro–pastoral ecotone of northern China, where precipitation is unevenly distributed in space and time and evaporation is intense. The long–term sustainability of planted sand–fixing vegetation depends largely on the balance between vegetation water consumption and environmental water supply. *Salix psammophila* is a native shrub widely used for windbreak and sand fixation and ecological restoration in the Mu Us Sandy Land because of its drought tolerance, strong sprouting ability, well–developed root system, and resistance to wind–blown sand [3,4]. As artificially planted *S. psammophila* stands develop, their branch structure, canopy characteristics, root distribution, and water demand may change, and stands of different ages may therefore exhibit different water–use characteristics [5,6]. Sap flow continuously reflects plant water transport and transpiration dynamics and is an important means of studying water use by woody plants in arid regions [7,8]. Previous studies have shown that environmental variables such as solar radiation, air temperature and humidity, soil moisture, wind speed, and vapor pressure deficit (VPD) are closely associated with plant sap flow [9,10]. Studies of *S. psammophila* have likewise shown clear diurnal and seasonal variation in sap flow, jointly influenced by hydrothermal conditions and precipitation events [11,12,13]. Recent studies have further revealed differences in sap flow among *S. psammophila* branches with different orientations [14], as well as the responses of daytime and nighttime transpiration water use to soil water availability during summer drought periods [15]. Nevertheless, most previous studies have focused on a single stand age or time scale, or have considered only a limited number of environmental variables. Systematic comparisons of sap flow characteristics and environmental response patterns among *S. psammophila* stands of different ages remain insufficient, particularly with respect to the combined effects of seasonal variation, temporal dependence, and age–related differences.

Studies of the relationships between sap flow and environmental factors have commonly used traditional statistical approaches, including Pearson correlation analysis, multiple linear regression, stepwise regression, and principal component analysis [9,10,12]. These methods are suitable for characterizing linear associations between variables; however, sap flow often co–varies with multiple environmental variables and may exhibit nonlinear responses, breakpoints, and variable interactions that cannot be fully characterized within a purely linear statistical framework. Random Forest (RF) can accommodate nonlinear relationships and multivariate interactions and has been applied to evapotranspiration estimation, hydrological prediction, and plant sap flow simulation [16,17,18,19]. SHapley Additive exPlanations (SHAP) can further quantify the relative contribution of each variable to model output and its directional changes [20,21]. Recent studies have applied interpretable machine–learning methods, such as SHAP, to quantitatively analyze the drivers of evapotranspiration in China [22]. Thus, combining conventional correlation analysis and segmented regression with RF and SHAP can characterize sap flow–environment relationships from both univariate statistical–association and multivariate–prediction perspectives. It should also be noted that continuous ecological observations commonly exhibit temporal autocorrelation and seasonal variation. Random sampling or out–of–bag evaluation may therefore be influenced by similarities among adjacent observations and may overestimate model applicability to unseen periods. Model validation should preserve temporal order and clearly distinguish correlation, variable importance, predictive performance, and causality. Accordingly, RF was used here primarily to identify the relative predictive contributions of environmental variables and nonlinear response patterns within the observation period, rather than as direct evidence for future sap flow prediction or physiological causal mechanisms.

Accordingly, this study investigated artificially planted *Salix psammophila* stands aged 3 years (3a), 8 years (8a), and 15 years (15a) in the Mu Us Sandy Land. Branch sap flow rates were continuously monitored from May to September 2025, together with environmental variables including solar radiation (Rs), air temperature (Ta), relative humidity (Rh), soil temperature (Ts), soil volumetric water content (Sh), wind speed (Ws), precipitation (Ra), and vapor pressure deficit (VPD). Sap flow dynamics analysis, Pearson correlation analysis, partial correlation analysis controlling for day of year (DOY), segmented regression, Random Forest (RF), SHAP interpretation, and cross–age model transfer evaluation were integrated to compare branch sap flow characteristics and their relationships with environmental variables among the sampled stands of different ages from the perspectives of temporal variation, univariate statistical associations, and multivariate prediction. Specifically, this study addressed the following three questions: (1) How do the diurnal and seasonal patterns of branch sap flow differ among *S. psammophila* stands of different ages? (2) What statistical associations and nonlinear response patterns exist between branch sap flow and soil moisture, temperature, and atmospheric evaporative conditions across stands of different ages, and to what extent are these relationships influenced by seasonal progression and temporal dependence? (3) How does the relative predictive information contained in environmental variables differ among stand–age–specific models, how well do these models transfer across stand ages, and to what extent are the sap flow–environment association patterns similar among stands? The results are intended to characterize temporal variation in branch–scale water transport and its environmental associations among *S. psammophila* stands of different ages in the study area, provide observational evidence for understanding water–use characteristics across different stages of stand development, and offer a reference for water management, stand management, and the sustainability assessment of ecological restoration in artificially planted sand–fixing shrublands of the Mu Us Sandy Land.

## 2. Results and Analysis

### 2.1. Dynamic Characteristics of Environmental Factors During the Observation Period

**Figure 1:**
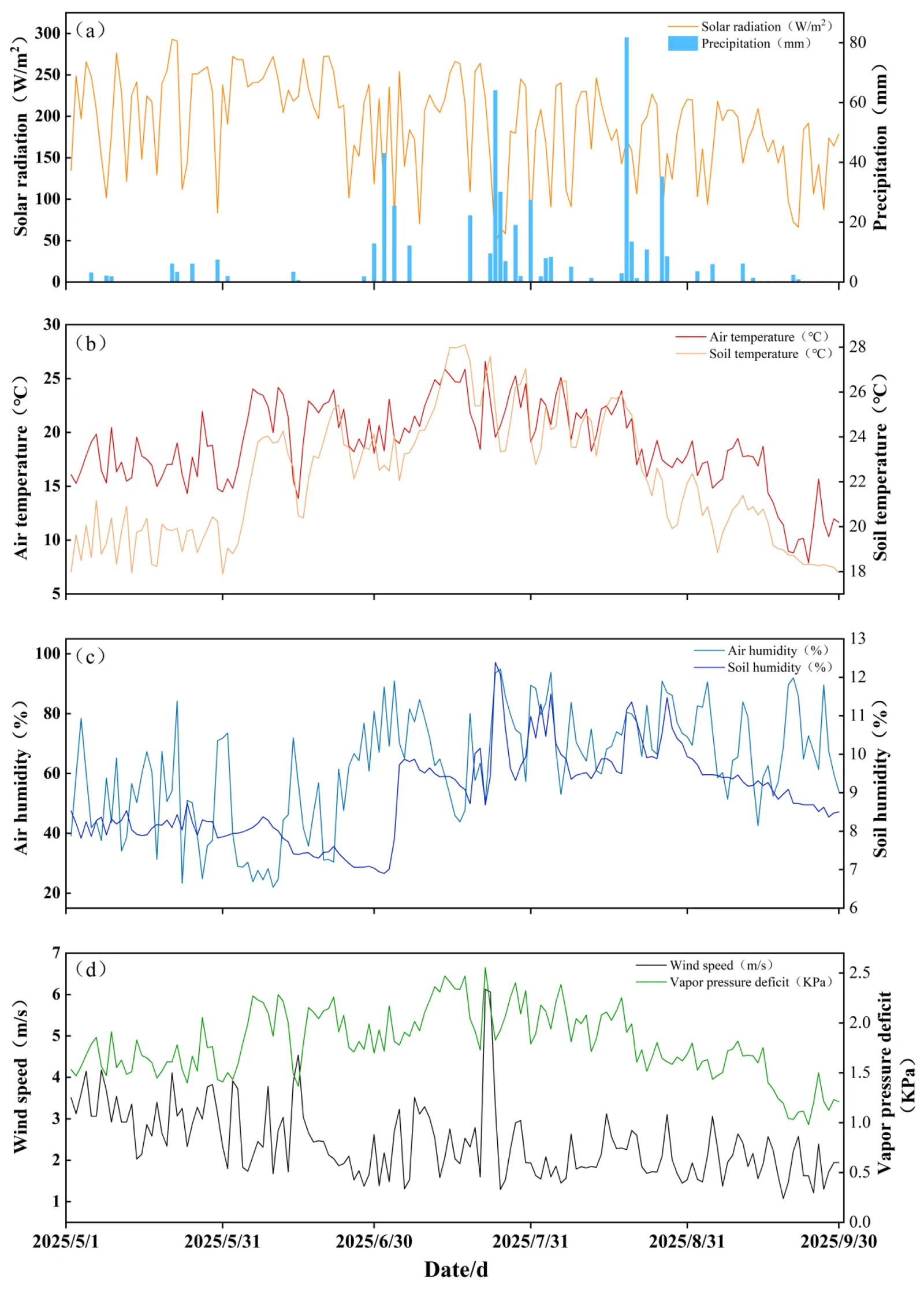
Seasonal dynamics of environmental and meteorological factors during the observation period (May–September). Note: (**a**) Rs and daily Ra; (**b**) Ta and Ts; (**c**) Rh and Sh; (**d**) Ws and VPD. The Ra bars represent daily precipitation; 515.21 mm is the cumulative value over the observation period obtained by summing daily Ra from May to September 2025.

During May–September 2025, all environmental variables showed pronounced seasonal dynamics (Figure 1, Table 1). Daily mean air temperature (Ta) generally increased and then decreased, with maximum and minimum daily mean values of 26.60 °C and 7.89 °C, respectively. The daily means of relative humidity (Rh), soil temperature (Ts), soil volumetric water content (Sh), vapor pressure deficit (VPD), solar radiation (Rs), and wind speed (Ws) were 62.22%, 22.15 °C, 8.91%, 1.79 kPa, 190.07 W/m^2^, and 2.42 m/s, respectively. Cumulative precipitation (Ra) from May to September 2025 was 515.21 mm, calculated by summing daily precipitation. Cumulative precipitation in July was 262.80 mm, accounting for 51.01% of precipitation during the observation period. The maximum daily precipitation was 81.80 mm on 19 August.

### 2.2. Temporal Dynamics of Sap Flow Rates in Salix psammophila Stands of Different Ages

Monthly mean sap flow rates of *S. psammophila* stands of different ages showed clear seasonal variation (Table 2). In May and September, branch–scale sap flow generally followed the order 15a > 8a > 3a, whereas from June to August the order shifted to 8a > 3a > 15a, indicating pronounced seasonal dependence in differences among stand ages. Throughout the observation period, sap flow rates in all age classes exhibited marked temporal fluctuations. The 15a stand reached its maximum value earliest, on 7 May (0.0272 kg·h^−1^); the 3a stand reached its maximum on 22 June (0.0246 kg·h^−1^); and the 8a stand reached its maximum on 17 August (0.0269 kg·h^−1^). In terms of monthly means, the 3a stand had its highest sap flow rate in June (0.0209 kg·h^−1^), whereas the 8a and 15a stands reached their highest monthly means in May, at 0.0222 and 0.0247 kg·h^−1^, respectively. Over the entire observation period, the 8a stand had the highest mean branch sap flow rate (0.0196 kg·h^−1^), followed by the 3a stand (0.0173 kg·h^−1^), with the 15a stand showing the lowest value (0.0168 kg·h^−1^).

**Figure 2:**
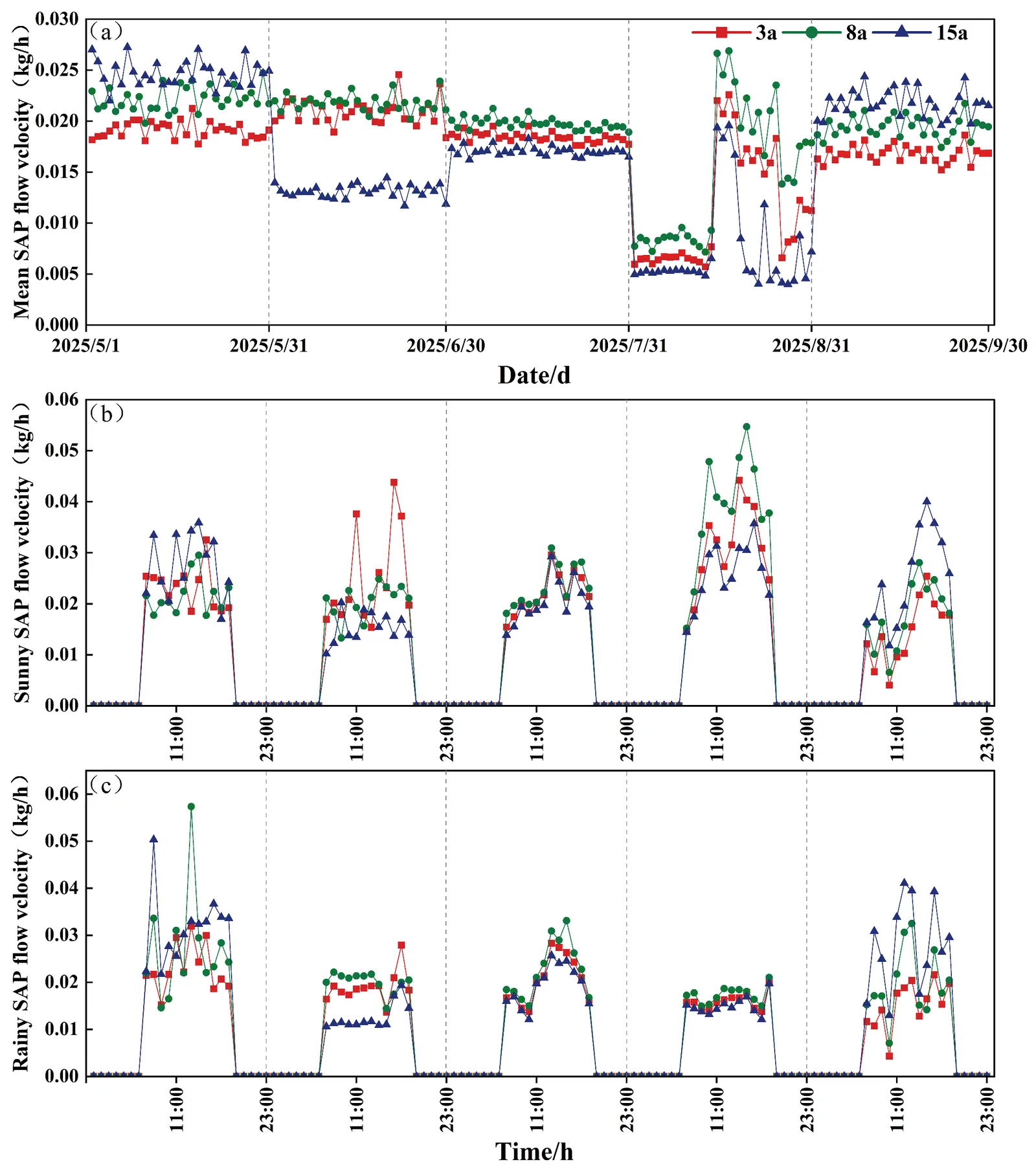
Dynamic characteristics of Q in *Salix psammophila* with different ages during the observation period. Note: (**a**) Daily average Q of *Salix psammophila* with different ages during the observation period. (**b**) Hourly average Q of *Salix psammophila* with different ages on a typical sunny day. (**c**) Hourly average Q of *Salix psammophila* with different ages on a rainy day.

Sap flow rates of *S. psammophila* stands of different ages exhibited clear diurnal rhythms (Figure 2). Sap flow generally began to increase at approximately 07:00, peaked around 14:00, and gradually declined to low levels after 19:00. Under sunny conditions, sap flow rates were generally higher than on rainy days, daytime values exceeded nighttime values, and a single–peaked diurnal pattern was observed. During midday (approximately 11:00–15:00), stronger solar radiation and higher air temperature coincided with higher sap flow rates, and peak sap flow in the 8a stand exceeded 0.05 kg·h^−1^; at night, sap flow declined to nearly 0.00 kg·h^−1^. Under rainy conditions, sap flow still followed a single–peaked diurnal pattern, but overall levels were lower, peak values were markedly reduced compared with sunny conditions and generally remained below 0.04 kg·h^−1^, and daytime fluctuations were smaller and more gradual. These changes coincided with lower solar radiation and higher air humidity. Sap flow patterns were broadly similar among stand ages under both sunny and rainy conditions, although the 8a stand showed relatively higher peak branch sap flow under both weather conditions, indicating a higher absolute sap flow level in its monitored branches in these two representative weather cases.

### 2.3. Correlation Analysis of Q and Environmental Factors

Autocorrelation analysis showed clear temporal dependence in the daily mean sap flow series of all stand ages (Table 3). First–order autocorrelation coefficients were 0.856, 0.823, and 0.906 for 3a, 8a, and 15a, respectively, and Ljung–Box tests were significant in all cases (*p* < 0.001). Pearson correlations between sap flow rate and environmental factors are shown in Table 4. Over the entire observation period, sap flow in the 3a stand was highly significantly positively correlated with Rs and Ws (*p* < 0.01) and highly significantly negatively correlated with Rh and Sh (*p* < 0.01), with r = −0.566 for Sh. In the 8a stand, sap flow was positively correlated with Rs, Ts, and Ws and negatively correlated with Rh and Sh, with r = −0.481 for Sh. In the 15a stand, sap flow was highly significantly positively correlated with Ws (r = 0.288, *p* < 0.01) and highly significantly negatively correlated with Ta, Ts, Rh, Sh, and VPD (*p* < 0.01), with r values of −0.386, −0.515, −0.211, −0.367, and −0.381, respectively. Monthly correlations showed pronounced temporal variation. In the 3a stand, sap flow was significantly positively correlated with Rs and negatively correlated with Ra in June, positively correlated with Ws in August, and highly significantly negatively correlated with Rh in September. In the 8a stand, sap flow was significantly positively correlated with Rs and negatively correlated with Ra in July, positively correlated with Ws in August, and highly significantly negatively correlated with Rh in September. In the 15a stand, sap flow was significantly positively correlated with Rs in July, positively correlated with Ts and Ws in August, and negatively correlated with Rh in September. After controlling for DOY, the previously significant negative correlations of 15a sap flow with Ta, Ts, and VPD were no longer significant (*p* > 0.05); Ws retained a weak positive correlation (r = 0.165, *p* < 0.05), and Sh retained only a weak negative correlation (r = −0.136). These results indicate that correlations between sap flow and some environmental variables in the 15a stand at the whole–growing–season scale were strongly season–dependent.

**Figure 3:**
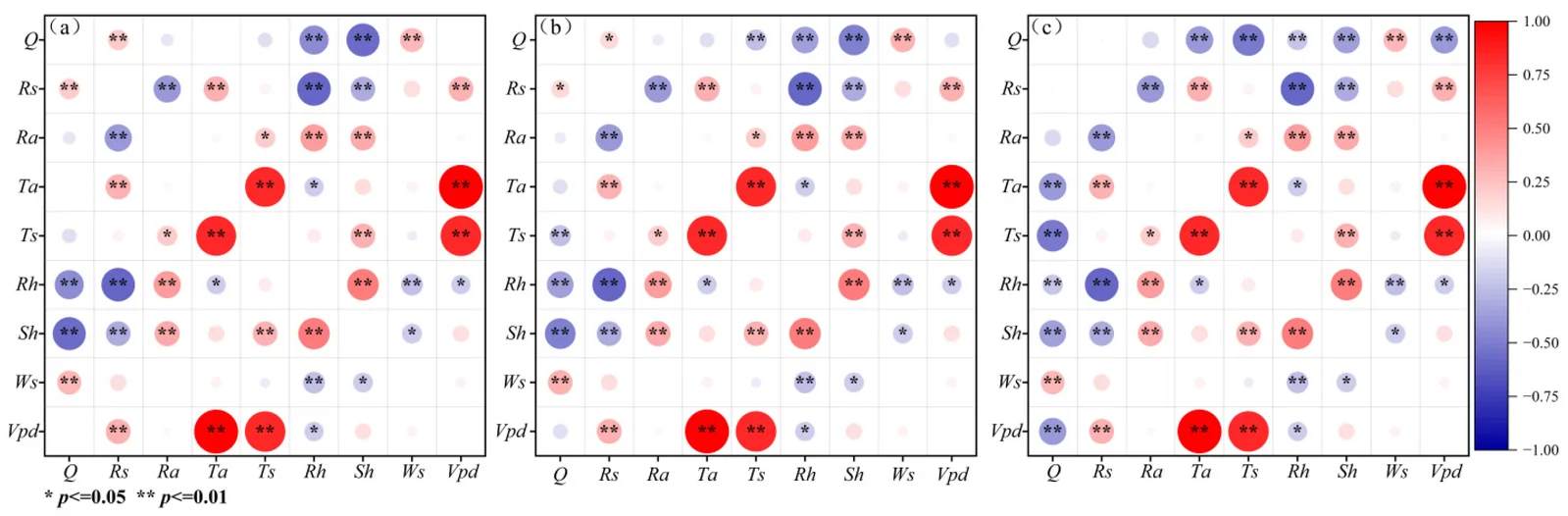
Correlation heatmap between Q and main environmental factors of *Salix psammophila* with different ages during the observation period. Note: (**a**) 3a *Salix psammophila*, (**b**) 8a *Salix psammophila*, (**c**) 15a *Salix psammophila*.

As shown in Figure 3, the correlations between the monthly mean Q and environmental factors differed somewhat among *S. psammophila* of different ages. For 3a, Q was generally positively correlated with Rs and Ws and negatively correlated with Rh and Sh; for 8a, Q was positively correlated with Rs and Ws and negatively correlated with Rh, Sh, and Ts; for 15a, Q was positively correlated with Ws and negatively correlated with Rh, Sh, Ta, Ts, and VPD. The environmental variables also showed marked correlations among themselves: Rs was positively correlated with Ta and VPD and negatively correlated with Ra, Rh, and Sh; Ra was generally positively correlated with Rh and Sh; Ta was positively correlated with Ts and VPD and negatively correlated with Rh; Rh was positively correlated with Sh and negatively correlated with Ws and VPD. Overall, sap flow and environmental variables showed certain co–variation characteristics at the monthly scale, but because the monthly analysis contains only five monthly observations, these results are mainly used to describe monthly variation trends and are not used as a basis for independent statistical inference or causality.

### 2.4. Responses of Q at Different Forest Ages to Major Environmental Factors

**Figure 4:**
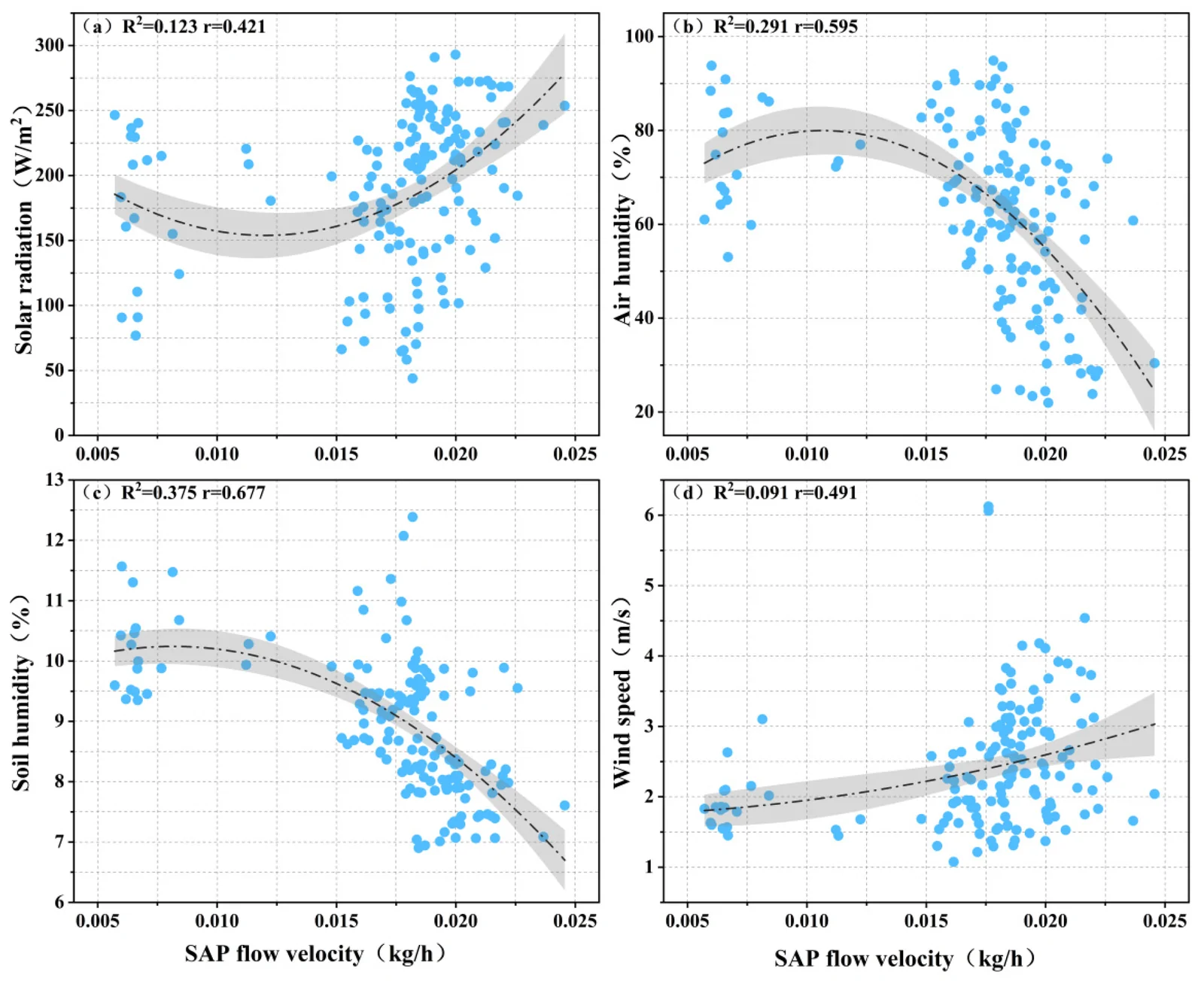
Response of Q in 3a *Salix psammophila* to major environmental factors. Note: R^2^ is the coefficient of determination for the linear fit, and r is the correlation coefficient; the gray area indicates the range of error in the fit results; the same applies below.

The nonlinear relationships between sap flow rate in the 3a stand and environmental factors are shown in Figure 4. The univariate linear–fit R^2^ values were, in descending order, Sh (0.375) > Rh (0.291) > Rs (0.123) > Ws (0.091). Segmented regression identified several statistical response transitions within the observed range, at approximately 150 W/m^2^ for Rs, 80% for Rh, 10% for Sh, and 2 m/s for Ws. Above the corresponding breakpoints for Rs and Ws, sap flow generally tended to increase, whereas below the corresponding values for Rh and Sh, the magnitude of change in sap flow increased.

**Figure 5:**
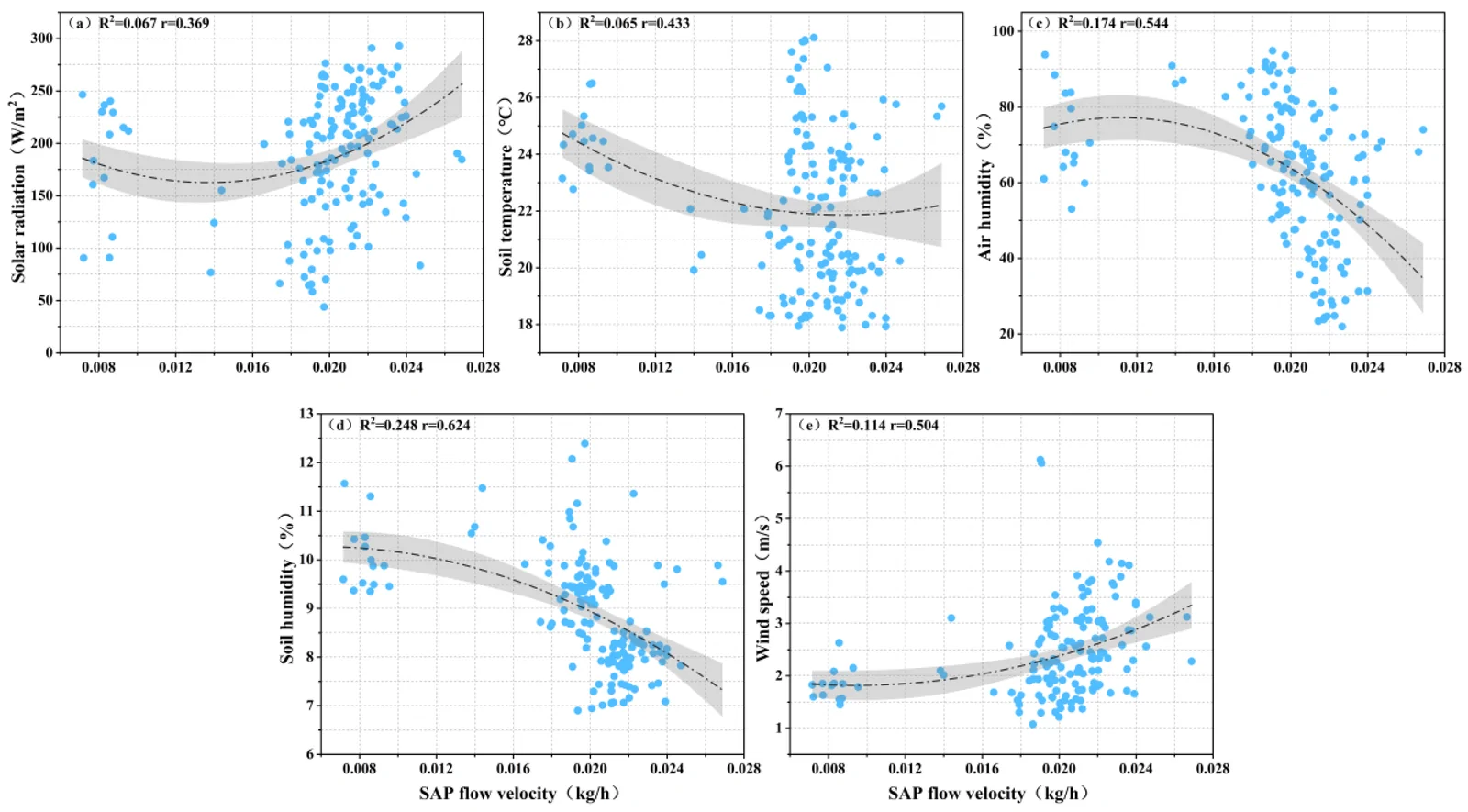
Responses of Q of 8a *Salix psammophila* to main environmental factors.

The nonlinear relationships between sap flow rate in the 8a stand and environmental factors are shown in Figure 5. The univariate linear–fit R^2^ values were Sh (0.248) > Rh (0.174) > Ws (0.114) > Rs (0.067) > Ts (0.065). Segmented regression identified statistical response transitions at approximately 150 W/m^2^ for Rs, 22 °C for Ts, 80% for Rh, 10% for Sh, and 2 m/s for Ws. Slopes or response magnitudes differed markedly on either side of the corresponding breakpoints, indicating nonlinear relationships.

**Figure 6:**
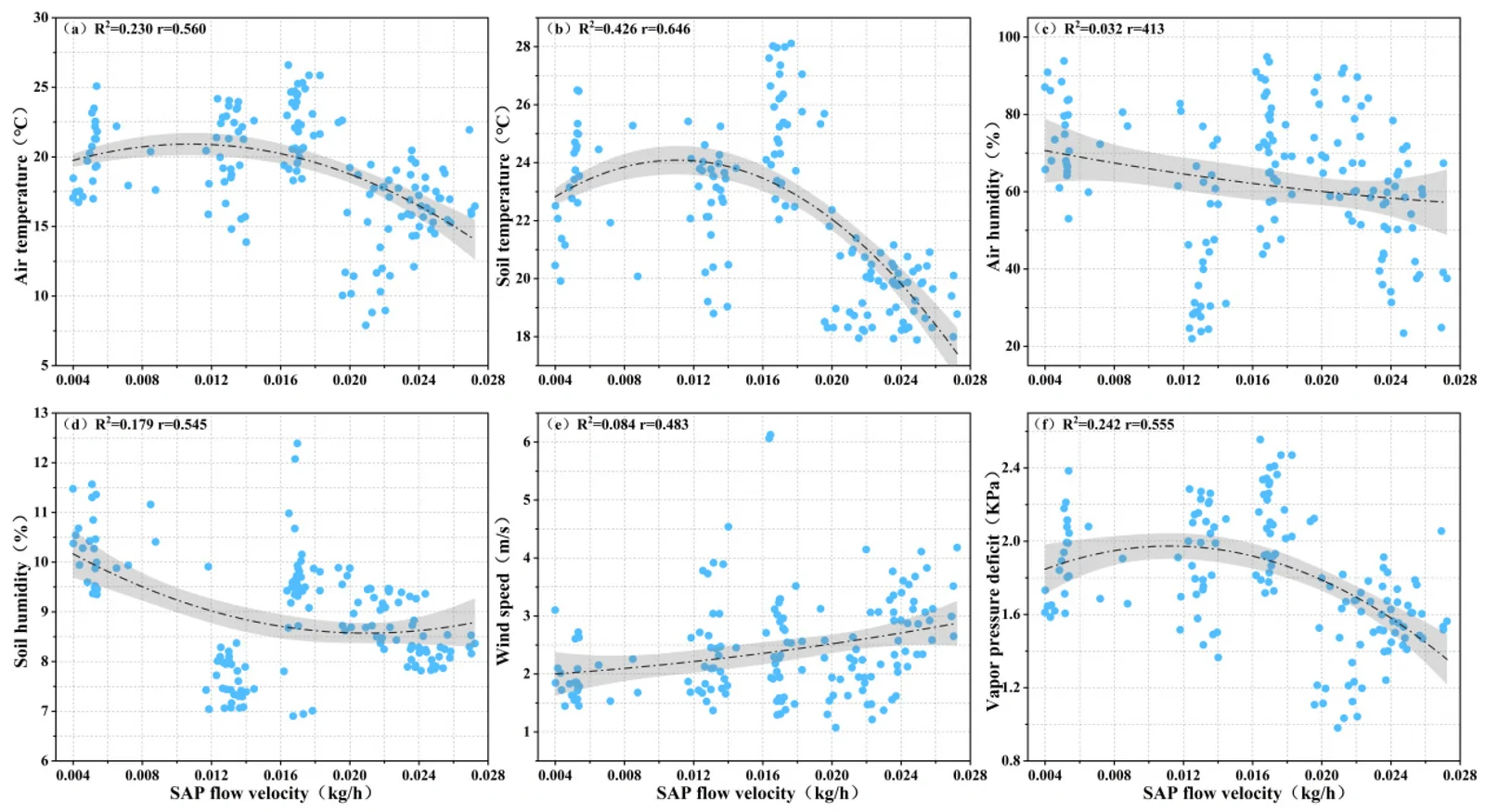
Responses of Q of 15a *Salix psammophila* to main environmental factors.

The nonlinear relationships between sap flow rate in the 15a stand and environmental factors are shown in Figure 6. The univariate linear–fit R^2^ values were Ts (0.426) > VPD (0.242) > Ta (0.230) > Sh (0.179) > Ws (0.084) > Rh (0.032), with Ts showing the highest statistical explanatory power. Segmented regression identified statistical response transitions at approximately 21 °C for Ta, 24 °C for Ts, 2.0 kPa for VPD, 70% for Rh, 9% for Sh, and 2.5 m/s for Ws. When VPD and Ta exceeded their corresponding breakpoints, sap flow generally declined; for Ts and Ws, the direction of the slope or magnitude of change differed across the corresponding breakpoints, indicating distinct nonlinear statistical associations between sap flow in the 15a stand and different environmental variables.

### 2.5. Assessment of Environmental Factor Importance and Model Interpretation Using Random Forests

**Figure 7:**
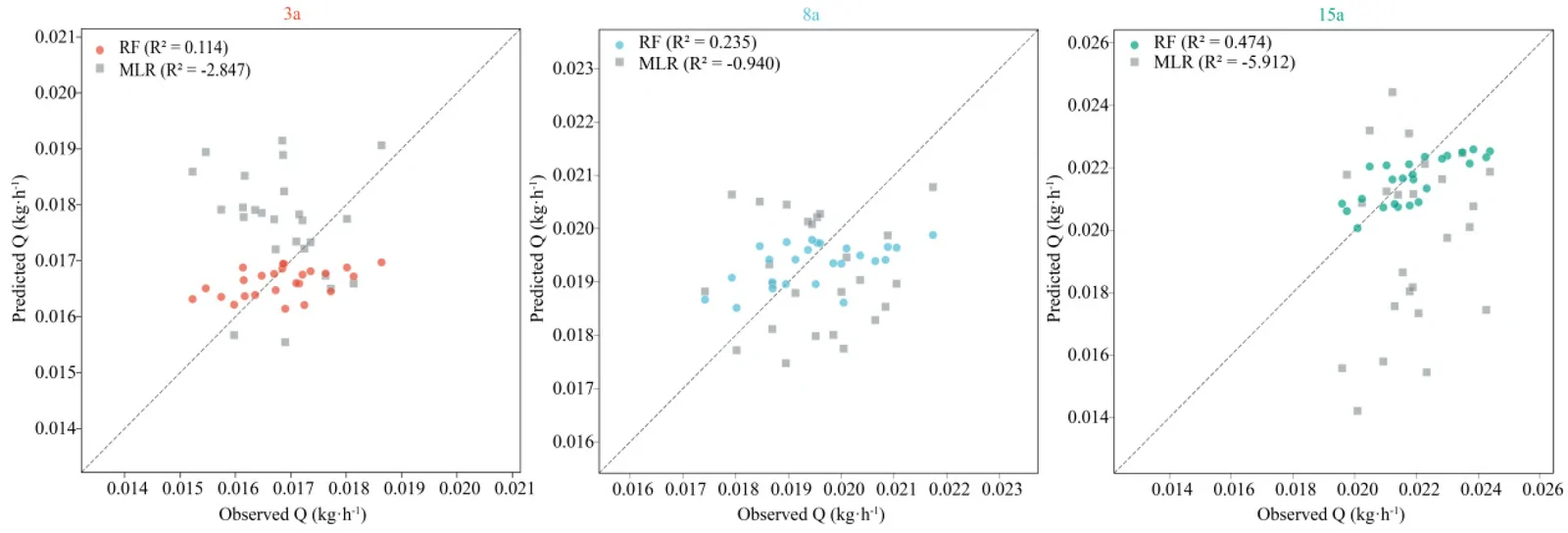
Scatter plot comparing the sap flow prediction accuracy of the RF and MLR models.

After nested grid–search optimization, the OOB–R^2^ values of Model A for 3a, 8a, and 15a were 0.343, 0.303, and 0.526, respectively, and the corresponding OOB–KGE values were 0.347, 0.304, and 0.617, indicating some ability to fit samples within the observation period. Because OOB sampling does not preserve strict temporal order, these estimates may be optimistically influenced by temporal autocorrelation and seasonal trends. After 5–fold forward–chaining time–series cross–validation, the mean CV–R^2^ values of the 3a, 8a, and 15a RF models were −1.371, −0.794, and −2.563, respectively, and the corresponding mean CV–KGE values were −0.262, −0.316, and −0.150. All were below 0 and varied substantially among folds, indicating limited and unstable temporal extrapolation to subsequent unseen periods. In particular, although the 15a model had the highest OOB–R^2^, it had the lowest CV–R^2^, showing that within–observation–period fit does not represent stable temporal generalization. Using the same eight environmental variables and identical temporal splits, both RF and MLR yielded negative CV–R^2^ values (Figure 7). RF outperformed MLR for 8a and 15a but performed worse than MLR for 3a, indicating that the temporal–generalization advantage of RF over a linear model was not consistent. The sensitivity model including previous–day sap flow rate Q_lag1 (Model B) also yielded negative CV–R^2^ values under the same validation framework: −0.812, −0.872, and −1.686 for 3a, 8a, and 15a, respectively. Thus, adding temporal–continuity information did not substantially improve temporal extrapolation. Accordingly, OOB metrics are not treated as evidence of strong predictive ability, and RF is not used for future sap flow prediction; instead, RF, SHAP, and PDP are used mainly to analyze the relative predictive contributions of environmental variables and nonlinear response patterns within the observation period. Overall model performance is summarized in Table 4, and fold–specific cross–validation results are provided in Table A1.

**Table 4:** Within–Observation–Period Fit and Temporal Cross–Validation Performance of Random Forest and Multiple Linear Regression Models.

Stand Age/a	OOB–R^2^	OOB–KGE	RF CV–R^2^	RF CV–KGE	MLR CV–R^2^	MLR CV–KGE
3a	0.343	0.347	−1.371 ± 1.422	−0.262 ± 0.181	−0.822 ± 0.453	−0.050 ± 0.166
8a	0.303	0.304	−0.794 ± 0.732	−0.316 ± 0.309	−3.501 ± 4.402	−0.291 ± 0.373
15a	0.526	0.617	−2.563 ± 2.361	−0.150 ± 0.123	−7.942 ± 6.304	−0.889 ± 0.732

Note: OOB–R^2^ and OOB–KGE describe the within–observation–period fit of RF models; CV–R^2^ and CV–KGE evaluate temporal extrapolation under 5–fold forward–chaining time–series cross–validation. RF and MLR use the same eight environmental factors and identical temporal splits. NSE and R^2^ are numerically identical in this study, and therefore only R^2^ is reported. CV–R^2^ < 0 indicates that model prediction error for an unseen period exceeds the error obtained using the mean observed value of that validation period as the baseline prediction.

**Figure 8:**
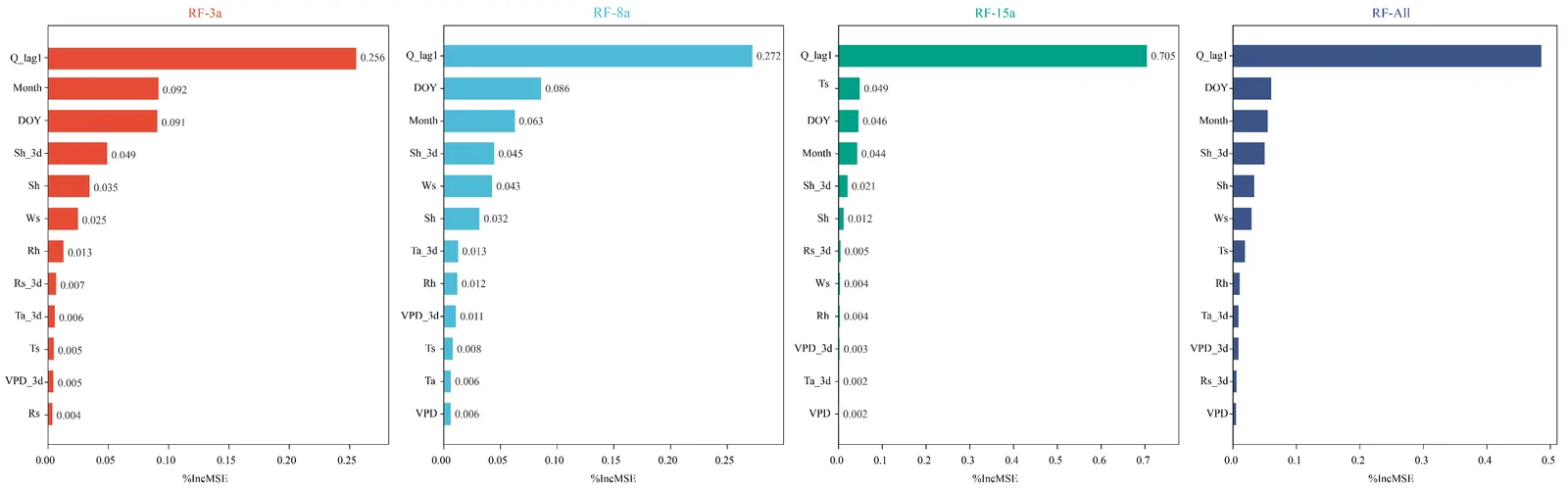
RF permutation importance: relative predictive contributions of environmental variables in the multivariate model.

**Figure 9:**
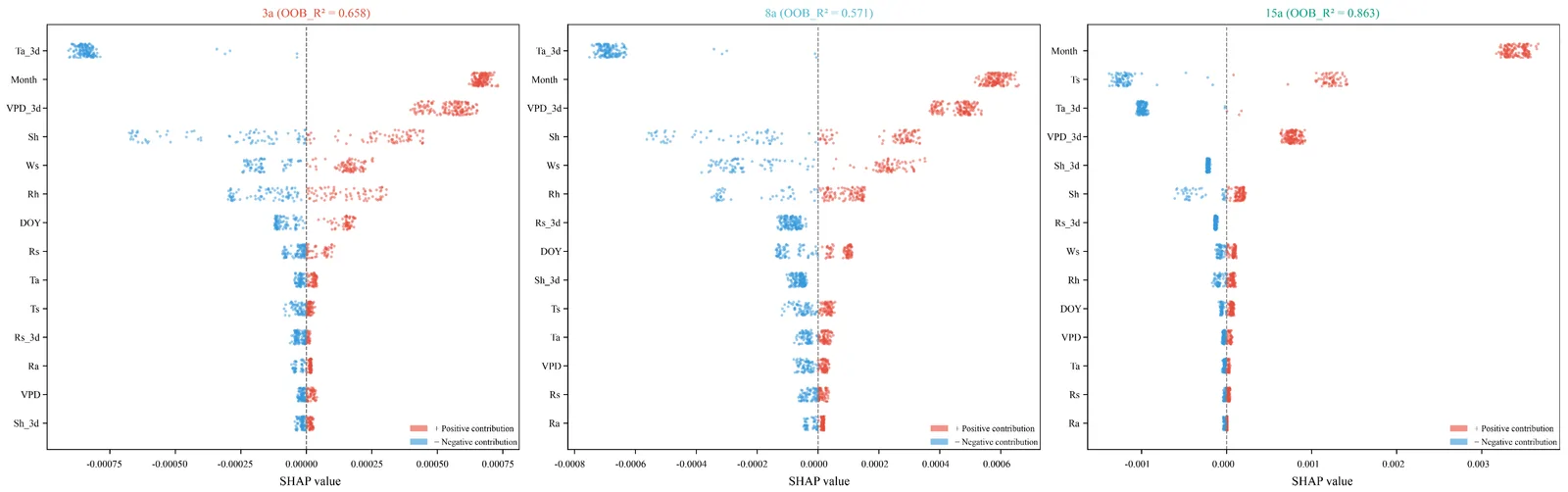
SHAP swarm plot: direction and magnitude of the relative contribution of each variable to model output.

**Figure 10:**
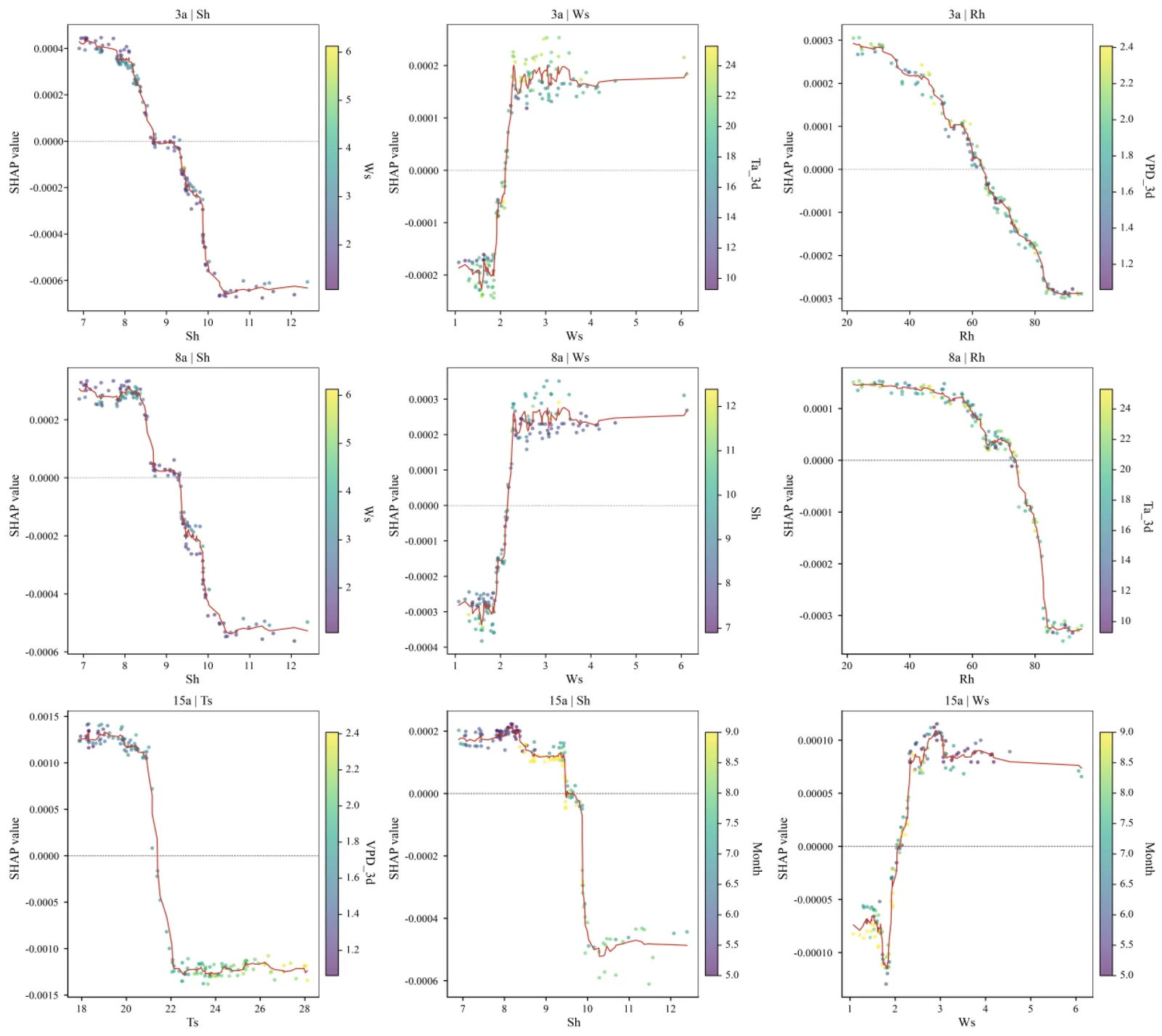
SHAP dependence plots: nonlinear statistical relationships between environmental variables and model output.

**Figure 11:**
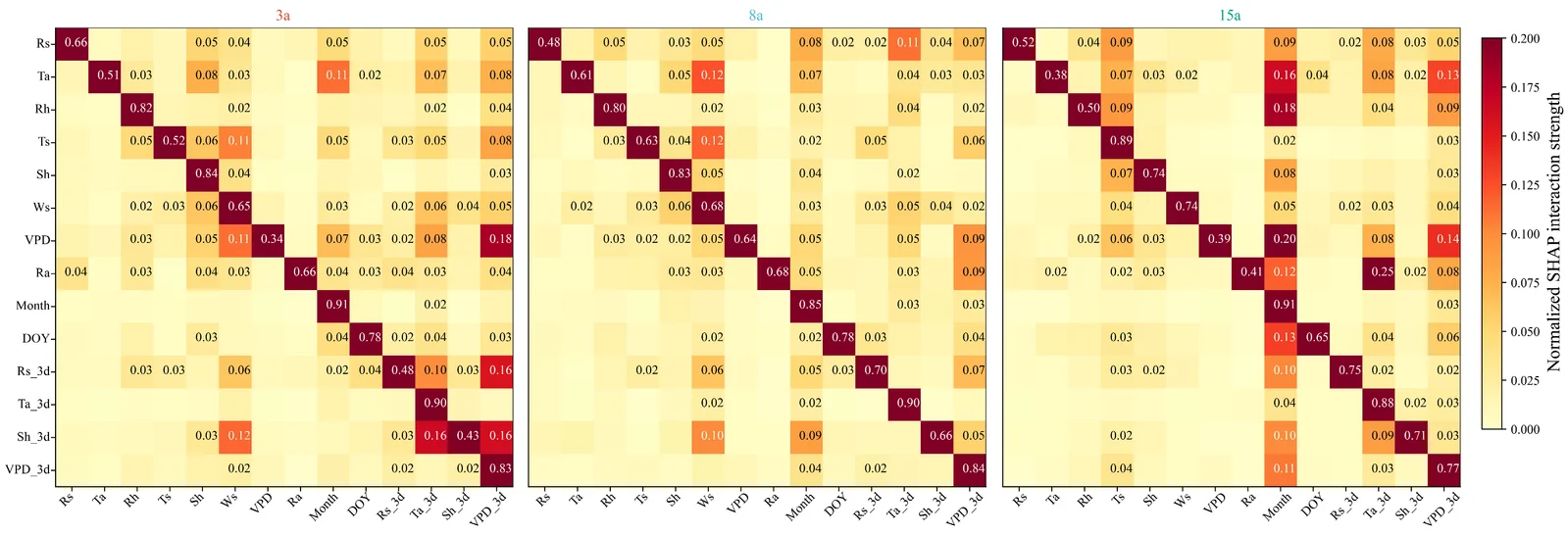
SHAP interaction matrix: model interaction information among environmental variables.

RF permutation importance and SHAP analyses showed that the relative predictive information contained in environmental variables differed among stand–age–specific models (Figure 8, Figure 9, Figure 10 and Figure 11). In the temporal–dependence sensitivity model containing Q_lag1, Q_lag1 had a relatively high predictive contribution in the 8a and 15a models, indicating that previous–day sap flow carried substantial predictive information for current model output. Among environmental variables, Sh, Ws, and Rh had relatively high predictive contributions in the 3a and 8a models, whereas Ts had the highest relative predictive contribution in the 15a model, followed by Sh and Ws. Because Ta, Ts, VPD, Rh, and Rs were correlated and shared seasonal variation, the above variable–importance and SHAP contributions represent relative predictive information within the current model and observation–data structure and should not be interpreted as independent effects of individual environmental factors. Correlated variables may share predictive information, making importance rankings model–dependent. SHAP swarm and dependence plots showed clear nonlinear statistical relationships between environmental variables and model output. Sh showed a response transition of approximately 8–12% in all three stand–age models; in the 15a model, Ts showed a model–response transition around 22 °C, above which its SHAP contribution generally increased. The SHAP interaction matrix showed relatively strong model interaction information between VPD and temperature variables in the 15a model; this result reflects statistical interaction information used by the model and is not direct evidence of physiological synergistic regulation.

**Figure 12:**
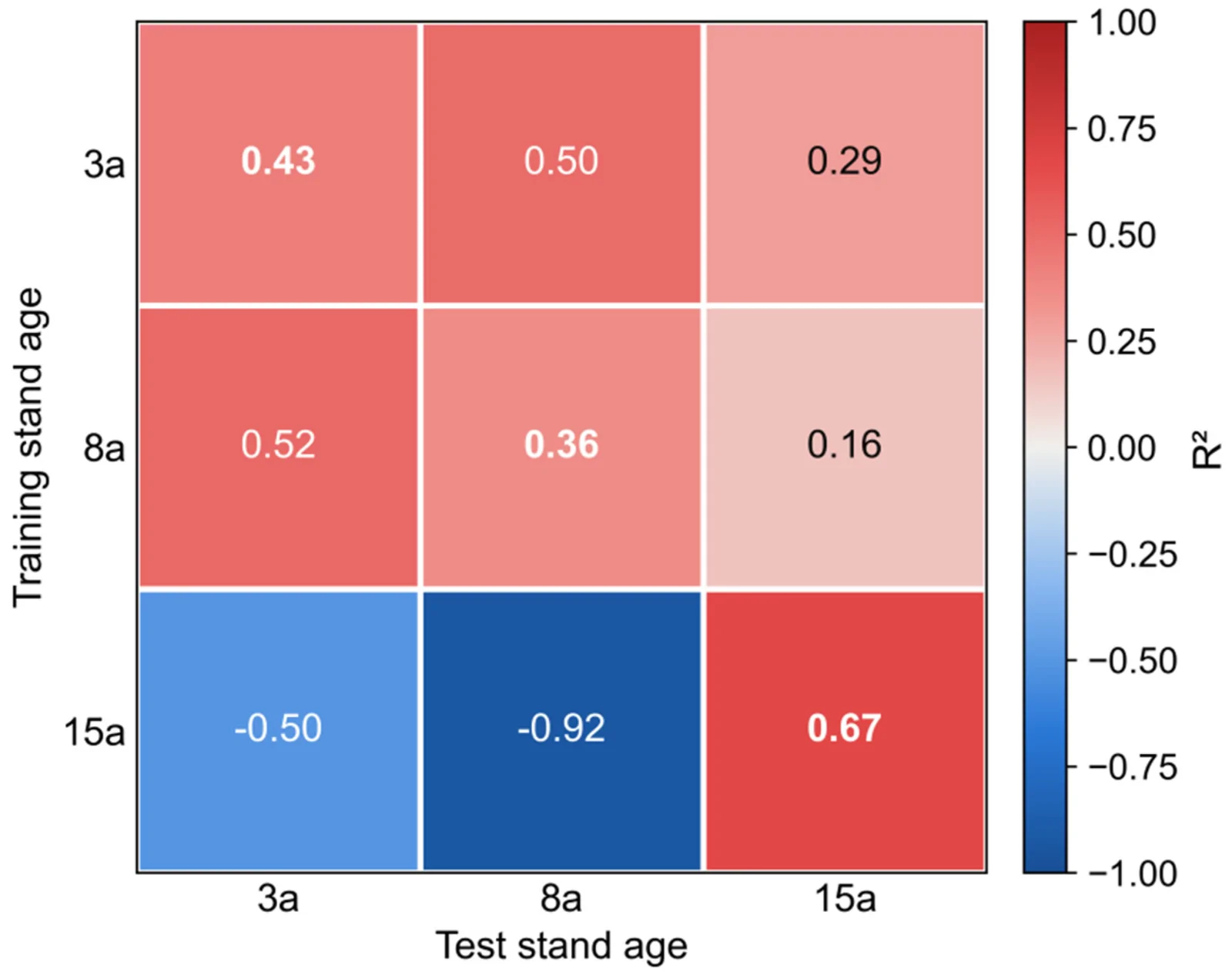
Validation matrix for cross–age–class generalization of the RF model.

The cross–age transfer evaluation (Figure 12) showed marked differences in model fit when models developed for one stand age were applied to data from other stand ages. The diagonal values in Figure 12 are the OOB–R^2^ values of Model A for each age class: 0.343, 0.303, and 0.526 for 3a, 8a, and 15a, respectively. Because these metrics use data partitions and validation objectives different from those of the OOB–R^2^ and forward CV–R^2^ reported in Table 5, they should not be directly compared or described as temporal–validation performance. In cross–age transfer, the transfer R^2^ values between the 3a and 8a models were 0.23 and 0.28, whereas the 15a model transferred to 3a and 8a yielded R^2^ values of −0.43 and −0.78, respectively. These results indicate that a model developed for one stand age has different levels of applicability to other stand–age data. The higher transfer R^2^ between 3a and 8a reflects similarity in their observed sap flow–environment statistical relationships and does not imply better temporal generalization than the model’s own forward–validation performance. Overall, transfer fit between 15a and the 3a and 8a stands was relatively poor, suggesting that the sap flow–environment association pattern in the mature stand differed substantially from those in the younger stands.

## 3. Discussion

### 3.1. Patterns of Sap Flow Variation and Water Use Characteristics in Salix psammophila of Different Ages

Plant water–use characteristics reflect the long–term interactions of root architecture, canopy structure, physiological activity, and environmental conditions [23]. This study found that branch–scale sap flow rates in the three *S. psammophila* stands generally followed the order 8a > 3a > 15a during the observation period, indicating differences in branch water–transport characteristics. Similar nonlinear age–related patterns have been reported in arid–region woody plants such as *Populus euphratica* [6] and poplar [7], suggesting that plant water transport or consumption does not necessarily increase continuously with stand age but may be jointly influenced by plant structure, canopy transpiration demand, and hydraulic characteristics. *S. psammophila* stands of different ages may differ in root development, canopy structure, and hydraulic architecture, all of which could affect branch sap flow [24]. However, sapwood area, leaf area, root distribution, and xylem hydraulic traits were not measured in this study. Therefore, the observed differences primarily represent differences in branch–scale absolute sap flow rates among the three sampled stands and cannot be used to infer water–transport capacity per unit conducting tissue or a specific physiological mechanism.

The relative ranking of sap flow among stand ages also showed pronounced seasonal variation. In May and September, the overall order was 15a > 8a > 3a, whereas from June to August it shifted to 8a > 3a > 15a, indicating strong seasonal dependence in differences among stands. Combined precipitation in May and September was approximately 52 mm, markedly lower than the 262.8 mm recorded in July; nevertheless, the 15a stand maintained relatively high branch sap flow during these drier periods. Previous studies suggest that as desert shrubs develop, their root systems may become broader or deeper, potentially allowing access to water from different soil layers [25]. However, only volumetric soil water content at 10 cm depth was monitored here, and deep–soil moisture, vertical root distribution, and plant water sources were not measured. Therefore, the present data cannot determine whether the 15a *S. psammophila* actually used deeper water sources. From June to August, as precipitation increased and shallow–soil water conditions changed, branch sap flow in the 3a and 8a stands generally increased, with the 8a stand maintaining a relatively high level while the 15a stand remained lower. These seasonal patterns suggest that water–use characteristics may differ among developmental stages and may involve coordination among water acquisition, canopy transpiration demand, and hydraulic safety [11,26]. It should be emphasized that this interpretation is only a working hypothesis based on observations from a single growing season and does not represent a directly physiologically validated mechanism of a shift in water–use strategy.

Diurnal sap flow in *S. psammophila* stands of different ages generally followed a single–peaked pattern with a slight midday decline, consistent with the pattern reported by Huang et al. [26] but differing from the bimodal pattern observed by Hao et al. [12] under severe water deficit. The midday decline coincided with higher temperature and VPD, but because stomatal conductance and leaf water potential were not measured simultaneously, it cannot be directly attributed to stomatal closure or a clearly defined midday depression of transpiration. Considering seasonal sap flow variation together with environmental dynamics, soil moisture, temperature, and atmospheric evaporative demand may all be associated with changes in branch sap flow, although their relative roles are further influenced by seasonal progression and covariation among variables. Overall, this study identified differences among the sampled stands in mean branch sap flow, seasonal ranking, and diurnal patterns. However, because branch size was not standardized, observations covered only a single growing season, and each stand age was represented by only one independent stand, these differences cannot currently be generalized as universal stand–age effects. Future studies should include multi–year observations, additional independent stands of the same age, and measurements of sapwood area, leaf area, deep–soil moisture, root distribution, stomatal conductance, leaf water potential, and xylem hydraulic traits to further test water–use patterns and their potential mechanisms across stand ages.

### 3.2. Environmental Association Patterns of Sap Flow in Salix psammophila Stands of Different Ages

Pearson correlation analysis, partial correlation analysis controlling for DOY, and segmented regression indicated differences in sap flow–environment statistical relationships among the sampled stands of different ages. In the 3a stand, sap flow showed a relatively strong whole–growing–season correlation with Sh (r = −0.566), and statistical response breakpoints for Sh, Rs, Rh, and Ws were approximately 10%, 150 W/m^2^, 80%, and 2 m/s, respectively, indicating clear statistical associations between branch sap flow and both soil–water and atmospheric conditions [27]. However, the negative whole–growing–season relationship between Sh and sap flow may partly reflect common seasonal variation, because increased Sh during the rainy season generally coincided with lower solar radiation and higher air humidity. Thus, univariate correlations should be interpreted in the context of seasonal background. In the 8a stand, Sh, Rh, and Ts also showed statistical associations with sap flow, and overall branch sap flow was relatively high among the three stands, but the available observations were insufficient to identify a specific physiological mechanism. The 15a stand showed stronger seasonal dependence, with relatively high sap flow in late spring and early autumn and lower values in midsummer. At the whole–growing–season scale, sap flow was significantly negatively correlated with Ta, Ts, and VPD (r = −0.386, −0.515, and −0.381, respectively). After controlling for DOY, these relationships were no longer significant (*p* > 0.05); only a weak positive correlation with Ws (r = 0.165, *p* < 0.05) and a weak negative correlation with Sh (r = −0.136) remained. This indicates that the original relationships of 15a sap flow with some environmental variables largely reflected common seasonal variation rather than independent short–term effects of Ta, Ts, or VPD. In addition, the daily sap flow series showed clear temporal autocorrelation, indicating that adjacent observations were not fully independent. Therefore, Pearson correlations, partial correlations, and subsequent model interpretations are discussed primarily as statistical associations and are not used to infer independent environmental effects or physiological causality. Overall, sap flow–environment association patterns differed among the three sampled stands, but whether these differences were primarily caused by stand age itself requires validation using additional independent stands of the same age.

Segmented regression further showed nonlinear statistical relationships between branch sap flow and environmental variables such as Sh, Ts, and VPD in some stands, with response transitions occurring within particular ranges. Previous studies have shown that soil moisture, soil temperature, and VPD are closely associated with water transport in woody plants [24,28,29], and the statistical transitions identified here are therefore broadly consistent with current ecophysiological understanding. However, these transition positions were derived from statistical fits between sap flow and environmental variables. Root water potential, root hydraulic conductance, stomatal conductance, and leaf water potential were not measured concurrently. Thus, response transitions near specific values of Sh, Ts, and VPD represent only statistical intervals within the observed range where sap flow–environment relationships changed; they should not be treated as physiological thresholds or used to infer that specific processes such as root hydraulic limitation or stomatal regulation occurred. Overall, the results support the view that *S. psammophila* branch sap flow co–varies with multiple environmental factors, including soil volumetric water content, thermal conditions, and atmospheric evaporative demand, rather than being determined by any single variable. Their relative roles and potential interactions require further validation using direct physiological measurements [30].

### 3.3. Multifactor Nonlinear Associations of Sap Flow Based on Random Forest and SHAP

Pearson correlation and segmented regression mainly describe statistical associations between individual environmental variables and sap flow, whereas RF, SHAP, and PDP can further characterize the relative predictive information contained in multiple variables and their nonlinear relationships with model output under multivariate conditions [19]. In this study, Sh, Ws, and Rh had relatively high predictive contributions in the 3a and 8a models, whereas Ts had a relatively high predictive contribution in the 15a model. Because Ta, Ts, VPD, Rh, and Rs were correlated and shared seasonal variation, different variables may share or substitute for part of the predictive information. Consequently, RF permutation importance, SHAP values, and PDPs are model–dependent interpretations and should not be taken to imply that a highly ranked variable has an equivalent independent ecological effect or physiological role [19]. At the same time, within–observation–period fit and forward temporal validation of the RF models differed substantially. OOB–R^2^ primarily described within–observation–period fit, whereas forward CV–R^2^ was below 0 for all three stand–age models and varied strongly among folds, indicating limited temporal generalization of sap flow–environment statistical relationships to subsequent unseen periods. Thus, RF, SHAP, and PDP are used here mainly to interpret relative predictive contributions and multivariate statistical response patterns within the observation period, rather than to quantify independent effects of individual environmental factors or establish physiological causality.

SHAP analysis further showed a pronounced stage–dependent nonlinear relationship between Sh and model output. When Sh was below approximately 8%, the SHAP contribution changed relatively strongly with Sh; between approximately 8% and 12%, the model showed a response transition; and above approximately 12%, the marginal predictive contribution generally became more gradual. This indicates that the RF model learned a nonlinear Sh–sap flow relationship, but these ranges represent only model–response intervals and should not be interpreted as definitive physiological soil–moisture thresholds. Previous studies have shown close relationships between soil water conditions and sap flow in woody plants of arid regions [29], suggesting that rhizosphere water–supply limitation may intensify at low soil volumetric water content. However, because root water potential and root hydraulic conductance were not measured, this interpretation requires direct physiological evidence. In the 15a model, SHAP analysis showed a response transition in Ts at approximately 22 °C, whereas univariate segmented regression identified a statistical breakpoint around 24 °C. This difference may arise from the different analytical frameworks: segmented regression describes the overall statistical relationship between a single variable and sap flow, whereas SHAP reflects the marginal contribution of a variable to multivariate model predictions and is influenced by other variables and their correlation structure [20,21]. Therefore, 22 °C and 24 °C are better regarded as statistical response intervals identified by different approaches rather than definitive physiological thresholds. SHAP interaction analysis showed relatively strong model interaction information between VPD and temperature variables in the 15a model, but this reflects statistical information used by the model and does not directly demonstrate physiological synergy.

Cross–age transfer analysis showed that models developed for different stand ages differed in their applicability to data from other stand ages. Reciprocal transfer between the 3a and 8a models yielded R^2^ values of 0.23 and 0.28, whereas transfer of the 15a model to the 3a and 8a data yielded R^2^ values of −0.43 and −0.78, respectively. This suggests some similarity in sap flow–environment statistical relationships between the 3a and 8a observations, whereas the 15a observations differed more substantially. It should be noted that cross–age transfer R^2^, OOB–R^2^, and forward CV–R^2^ correspond to different data partitions and evaluation objectives and therefore cannot be directly compared or used to infer temporal generalization. Because each stand age was represented by only one stand, these transfer results primarily reflect differences among the three sampled stands and cannot yet be generalized as universal stand–age effects. Taken together, the RF, SHAP, and cross–age transfer results indicate differences in branch sap flow–environment association patterns among the sampled stands: variables such as Sh contained relatively more predictive information in the 3a and 8a models, whereas Ts and variables related to atmospheric evaporative conditions contributed relatively more predictive information in the 15a model, which also showed stronger seasonal dependence. Sap flow variation under different environmental conditions may be jointly influenced by soil moisture, temperature, and atmospheric evaporative demand [29,31], but the present results are insufficient to attribute these differences directly to an age–driven shift in water–use strategy. The underlying mechanisms require further testing using additional independent stands of the same age together with direct measurements of sapwood area, leaf area, root distribution, stomatal conductance, leaf water potential, and xylem hydraulic traits.

## 4. Materials and Methods

### 4.1. Study Area

The study area is located in the Maogaitu forestland of Operation Area 68, Tagemiao, Xinjie Desert Control Station, Yijinhuoluo Banner State–owned Forest Farm, Ordos City, Inner Mongolia Autonomous Region (39°10′30″ N, 109°35′47″ E), on the southeastern margin of the Mu Us Sandy Land. The region has a typical temperate semi–arid continental monsoon climate. The long–term mean annual temperature is approximately 9.7 °C, and the long–term mean annual precipitation is approximately 393 mm. According to long–term climatic records for the study area, precipitation is strongly seasonal and occurs mainly in summer, with approximately 60–75% of annual precipitation falling from July to September; atmospheric evaporative demand is also relatively high. The landscape is dominated by fixed and semi–fixed sand dunes, and the main soil type is aeolian sandy soil, characterized by high sand content, loose structure, relatively low organic matter and nutrient contents, high permeability, and low water–holding capacity. The major planted woody and herbaceous species in the region include *Salix psammophila*, *Caragana korshinskii*, *Artemisia ordosica*, and *Corethrodendron scoparium*. Among these species, *S. psammophila* is highly tolerant of drought and wind–blown sand and is one of the dominant species used in ecological restoration and windbreak and sand–fixation projects in the Mu Us Sandy Land [3].

### 4.2. Study Subjects and Plot Setup

From May to September 2025, artificially planted *S. psammophila* stands aged 3, 8, and 15 years were selected to represent different stages of stand development using a space–for–time substitution approach. Stand age was determined from local afforestation records. *Salix psammophila* is a sprouting shrub, and branches within a clump are mostly regenerated after coppicing; therefore, branch age does not necessarily correspond to stand age, and stand age was used as the basis for grouping. The study included three stands of different ages, with three 20 m × 20 m plots established within each stand, for a total of nine plots, to characterize spatial variation within the stands. It should be noted that the three plots within the same stand constitute a spatial sub–sample rather than independent replicates at the stand age level. Therefore, the interpretation of differences between different stand ages in this paper is primarily limited to the three stands surveyed in this study. The stands were selected to be as similar as possible in soil type, topography, slope aspect, groundwater depth, stand density, and management history to minimize the potential influence of non–age–related site factors. Within each plot, three healthy *S. psammophila* clumps were selected, and plant height, crown width, branch basal diameter, and branch number were measured. Plant height and crown width (east–west × north–south) were measured using a measuring tape, and branch basal diameter was measured using a vernier caliper; plant morphological characteristics are shown in Table 5 and Table A2. Two morphologically uniform, healthy, and undamaged branches were selected from each clump for sap flow monitoring at approximately 50 cm above ground level, giving 54 monitored branches in total. Branch diameters ranged from 3 to 15 mm.

**Table 5:** Basic Characteristics of *Salix psammophila* Plants of Different Ages (Mean ± Standard Deviation).

Stand Age/a	Branch Diameter/cm	Tree Height/m	Crown Width/m × m	Number of Branches/Clump
3a	0.48 ± 0.03	1.60 ± 0.45	1.86 × 1.64	28 ± 4
8a	0.59 ± 0.07	2.07 ± 0.58	2.31 × 1.94	42 ± 6
15a	0.61 ± 0.02	2.70 ± 0.10	2.51 × 2.25	35 ± 5

### 4.3. Monitoring of Sap Flow Rates in Twigs

An EMS62 wrap–around plant stem sap flow measurement system (Environmental Measuring Systems, Brno, Czech Republic), based on the Tissue Heat Balance (THB) method, was used to continuously monitor branch sap flow rates of *S. psammophila*. The system was suitable for the branch diameter range used in this study, and all monitored branches were measured using the same wrap–around installation method. Sensors were installed approximately 50 cm above ground level. Before installation, branch diameter was measured and the installation surface was cleaned to ensure close contact between the sensor and the branch. After installation, the sensor was secured according to the manufacturer’s instructions and an external protective cover was fitted to reduce the effects of solar radiation and environmental fluctuations on measurements. Raw signals were collected at 60–s intervals, 10–min mean sap flow rates were calculated automatically, and data were stored at 30–min intervals. The study included three stands of different ages, with one stand per age class, three plots per stand, three *S. psammophila* clumps per plot, and two monitored branches per clump, giving 54 monitored branches in total. The monitoring period extended from 1 May to 30 September 2025 (153 d). Eighteen branches were monitored for each age class, and daily mean sap flow rates were calculated from the 30–min measurements. Branch–scale absolute sap flow rate Q (kg·h^−1^) was used to characterize the mean water transport rate of the monitored branches. Because effective sapwood area and leaf area were not measured, Q was not standardized by sapwood area or leaf area. Absolute Q may therefore be influenced by branch size, water–conducting status, and other structural factors. Accordingly, differences in Q among stands of different ages are interpreted only as differences in branch–scale absolute sap flow rate and not as differences in physiological water–conducting capacity per unit conducting tissue.

### 4.4. Monitoring of Environmental Parameters

A BRS1000 Bowen ratio energy balance observation system was deployed in an open area of the study region to continuously monitor solar radiation (Rs, W/m^2^), precipitation (Ra, mm), wind speed (Ws, m/s), air temperature (Ta, °C), relative humidity (Rh, %), soil temperature (Ts, °C; 10 cm depth), and soil volumetric water content (Sh, %; 10 cm depth). The observation system was used primarily to characterize the common environmental background of the study area. Because independent meteorological instruments were not deployed in each plot, these environmental data could not represent plot–specific microclimatic differences. Data were recorded at 30 min intervals over the same period as sap flow monitoring (May–September 2025). Vapor pressure deficit (VPD, kPa) was calculated from air temperature and relative humidity [4]:

VPD = 0.6108 × exp (17.27Ta/(Ta + 237.3)) × (1 − Rh/100)(1)

where VPD is the vapor pressure deficit (kPa); Ta is the air temperature (°C); and Rh is the relative humidity of the air (%).

### 4.5. Data Processing and Statistical Analysis

Microsoft Excel 2021 (Microsoft Corporation, Redmond, WA, USA) was used to organize, screen, and quality–control the raw data. Invalid records were identified only on the basis of explicit data–acquisition failures, including sensor power loss, data–transmission interruptions, or instrument malfunction that prevented valid sap flow records from being obtained. Extreme weather itself was not used as an exclusion criterion; valid measurements recorded during extreme weather while the instruments were operating normally were retained. Daily–scale data were used as the principal analytical unit. Daily mean sap flow rate was calculated from valid 30–min observations for each day and expressed in kg·h^−1^; this metric represents the mean within–day water transport rate rather than cumulative daily sap flow. Daily Ta, Rh, Ts, Sh, Ws, Rs, and VPD were calculated as the means of the corresponding 30 min observations, whereas Ra was calculated as daily cumulative precipitation by summing precipitation across all intervals within each day. Pearson correlation analysis was used to assess the overall statistical associations between daily mean sap flow rate and Rs, Ta, Rh, Ts, Sh, Ws, Ra, and VPD for each stand age. Each age–class analysis included 153 consecutive daily time–series observations (*n* = 153) obtained from repeated measurements of the same monitored branches; thus, *n* = 153 represents the number of temporal observations rather than independent biological replicates. To assess temporal dependence, autocorrelation analysis was performed on the daily mean sap flow series for each stand age, and the Ljung–Box test was used to evaluate serial autocorrelation. Month–by–month analyses were used mainly to describe changes in sap flow–environment association patterns across months. Because monthly sample sizes and environmental ranges were limited, these analyses were not used for strong independent significance inference; the five monthly means were used only to describe intermonthly and seasonal trends.

Because sap flow and environmental variables such as Ta, Ts, and VPD exhibited pronounced seasonal variation, day of year (DOY) was used as a proxy for seasonal progression, and partial correlation analysis controlling for DOY was used to reassess daily–scale associations between sap flow and environmental factors while reducing the influence of common seasonal trends. Relationships whose significance markedly weakened or disappeared after controlling for DOY were interpreted only as statistical associations at the whole–growing–season scale and not as evidence of independent environmental effects or physiological causality. Nonlinear responses between sap flow and environmental variables were analyzed using segmented linear regression. Candidate breakpoints were searched at 1% percentile intervals, and the breakpoint minimizing the residual sum of squares was selected. When no clear single transition existed, LOESS (span = 0.3) was used to assist in describing the overall trend. Because uncertainty in breakpoint locations and slopes on either side was not independently estimated, the identified breakpoints were treated only as exploratory statistical response breakpoints or transition intervals within the observed range and not as physiologically validated functional thresholds. LOESS was used only for descriptive smoothing and did not contribute to breakpoint determination. All statistical tests were two–sided, with significance levels of α = 0.05 and α = 0.01.

### 4.6. Random Forest Modeling and SHAP Explainability Analysis

A Random Forest (RF) regression model was used to analyze nonlinear statistical relationships between environmental variables and sap flow rate, together with permutation importance, SHAP (SHapley Additive exPlanations), and partial dependence plots (PDPs) to interpret the relative predictive contributions of variables and their response patterns. The environmental explanatory model (Model A) included eight environmental variables: Rs, Ta, Rh, Ts, Sh, Ws, VPD, and Ra, and was fitted separately for each stand age. A sensitivity model (Model B) additionally included the previous day’s sap flow rate (Q_lag1) to test the influence of temporal–continuity information on model performance. Because variables such as Ta, Ts, VPD, Rh, and Rs were correlated and shared seasonal variation, variable importance, SHAP values, and PDPs were used only to describe relative predictive information and nonlinear statistical relationships under the current model and data structure, rather than independent effects or physiological causal roles of individual environmental variables.

For the continuous time–series data, 5–fold forward–chaining expanding–window cross–validation (Time Series Split, n_splits = 5) was used to evaluate temporal generalization. The 153 daily observations were ordered chronologically; each validation set comprised the subsequent 25 consecutive days, and the corresponding training sets contained 28, 53, 78, 103, and 128 d, respectively, ensuring that each validation period always followed its training period. Hyperparameters were optimized using nested forward time–series grid search performed exclusively within the training data of each fold; validation data were not used for parameter selection, thereby avoiding future–information leakage. The search space was n_estimators ∈ {200, 500, 1000}, max_depth ∈ {None, 5, 10, 20}, min_samples_leaf ∈ {1, 2, 5}, and max_features ∈ {0.33, 0.5, 1.0}. The optimization criterion was maximum inner–cross–validation R^2^, and the random seed was set to 42. The final Model A parameters were 3a (200, 10, 5, 0.33), 8a (200, 20, 5, 0.33), and 15a (1000, None, 5, 1.0), corresponding in order to n_estimators, max_depth, min_samples_leaf, and max_features. After temporal cross–validation, final interpretation models were refitted using the complete observation–period data with the same parameter–search strategy, and permutation importance, SHAP values, and PDPs were calculated from these models. These results were used to interpret relationships within the observation period rather than as validation results on independent held–out data.

Model performance was evaluated using the coefficient of determination (R^2^) and Kling–Gupta efficiency (KGE). OOB–R^2^ and OOB–KGE described within–observation–period fit of the RF models, whereas CV–R^2^ and CV–KGE were calculated from forward time–series cross–validation to evaluate temporal generalization to subsequent unseen periods. Multiple linear regression (MLR), using the same eight environmental variables and identical temporal splits, served as a benchmark model. Because Nash–Sutcliffe efficiency (NSE) used the same mean–based reference as R^2^ and yielded identical numerical values in this study, only R^2^ is reported in the main text. For cross–age transfer analysis, the final model from one stand age was directly applied to data from another stand age without parameter refitting. RF and SHAP analyses were implemented in Python 3.9 using scikit–learn 1.6 and SHAP 0.49.

## 5. Conclusions

Based on observations from a single growing season (May–September 2025), this study compared branch–scale absolute sap flow rates and their statistical associations with environmental variables among three *S. psammophila* stands aged 3a, 8a, and 15a in the Mu Us Sandy Land. During the observation period, Q generally followed the order 8a > 3a > 15a, although the seasonal ranking changed. Because each age class was represented by only one stand and monitored branch sizes differed, these differences primarily reflect branch–scale sap flow differences among the sampled stands and cannot be attributed solely to stand age or interpreted as hydraulic capacity per unit conducting tissue. In the 3a and 8a stands, Sh showed relatively strong whole–growing–season statistical associations with Q. In the 15a stand, significant negative correlations of Q with Ta, Ts, and VPD disappeared after controlling for DOY, indicating pronounced seasonal dependence. RF OOB metrics described only within–observation–period fit, whereas CV–R^2^ from 5–fold forward time–series cross–validation was below 0 for all models, indicating limited temporal extrapolation to subsequent unseen periods. Accordingly, RF, SHAP, and PDP were used only to interpret relative predictive information and nonlinear statistical relationships within the observed range. Breakpoints identified by segmented regression and SHAP were treated as exploratory statistical response positions rather than evidence of physiological thresholds or causal mechanisms. Future studies should include additional independent stands of the same age and multi–year observations, together with measurements of sapwood area, leaf area, multilayer soil moisture, water sources, stomatal conductance, leaf water potential, and xylem hydraulic traits, to test the stability and physiological basis of these patterns.

## Figures and Tables

**Table 1:** Monthly average change characteristics of environmental and meteorological factors during the observation period (May–September).

Month	Ta (°C)	Rh (%)	Ts (°C)	Sh (%)	VPD (kPa)	Rs (W/m^2^)	W_S_ (m/s)	Ra (mm/d)
May	17.10 ± 1.87	51.22 ± 15.45	19.45 ± 0.90	8.16 ± 0.23	1.62 ± 0.16	208.12 ± 59.98	3.13 ± 0.57	0.99 ± 2.09
June	20.41 ± 2.98	45.54 ± 18.23	22.74 ± 1.79	7.61 ± 0.41	1.92 ± 0.27	224.79 ± 44.40	2.44 ± 0.82	0.71 ± 2.43
July	22.34 ± 2.48	70.78 ± 14.51	25.19 ± 1.89	9.48 ± 1.21	2.10 ± 0.25	180.50 ± 72.07	2.56 ± 1.15	8.48 ± 15.51
August	20.23 ± 2.51	74.33 ± 9.83	23.65 ± 1.87	10.20 ± 0.66	1.90 ± 0.23	178.78 ± 47.87	2.01 ± 0.46	5.78 ± 15.76
September	14.36 ± 3.52	68.91 ± 13.30	19.66 ± 1.27	9.08 ± 0.41	1.42 ± 0.26	158.22 ± 44.31	1.93 ± 0.47	0.71 ± 1.67

Note: Ra, the monthly mean of daily precipitation (mean ± standard deviation).

**Table 2:** Monthly Statistical Characteristics of Sap Flow Rates in *Salix psammophila* Stands of Different Ages (Unit: kg·h^−1^).

Month	Stand Age/a	Minimum	Maximum	Mean	Standard Deviation
May	3a	0.0178	0.0212	0.0191	0.0008
	8a	0.0198	0.0247	0.0222	0.0012
	15a	0.0220	0.0272	0.0247	0.0013
June	3a	0.0184	0.0246	0.0209	0.0013
	8a	0.0202	0.0239	0.0218	0.0008
	15a	0.0117	0.0145	0.0131	0.0006
July	3a	0.0176	0.0195	0.0184	0.0005
	8a	0.0189	0.0212	0.0197	0.0006
	15a	0.0162	0.0183	0.0170	0.0005
August	3a	0.0057	0.0226	0.0113	0.0057
	8a	0.0072	0.0269	0.0147	0.0067
	15a	0.0040	0.0196	0.0073	0.0047
September	3a	0.0152	0.0186	0.0168	0.0008
	8a	0.0174	0.0217	0.0194	0.0010
	15a	0.0196	0.0244	0.0217	0.0013

**Table 3:** Daily variation correlation between sap flow and main environmental factors of *Salix psammophila* with different ages.

Month	Stand Age/a	Rs	Ra	Ta	Ts	Rh	Sh	Ws	VPD
May	3a	−0.185	0.107	−0.148	−0.117	−0.222	−0.115	0.147	−0.149
	8a	−0.119	0.417 *	−0.102	−0.034	0.020	0.031	0.245	−0.101
	15a	−0.163	−0.064	−0.104	−0.175	−0.218	0.104	0.025	−0.096
June	3a	0.423 *	−0.395 *	0.0147	−0.030	−0.285	0.077	0.062	0.158
	8a	0.187	−0.087	−0.081	0.069	−0.039	0.077	0.056	−0.079
	15a	0.041	−0.263	−0.104	−0.204	0.107	−0.166	0.137	−0.087
July	3a	0.368 *	−0.334	0.081	−0.183	−0.196	−0.279	−0.307	0.080
	8a	0.367 *	−0.356 *	0.127	−0.125	−0.224	−0.191	−0.175	0.123
	15a	0.389 *	−0.322	0.244	0.000	−0.269	−0.091	−0.292	0.242
August	3a	0.006	0.078	−0.029	0.129	−0.123	−0.062	0.428 *	−0.028
	8a	−0.028	0.104	−0.161	−0.044	−0.051	0.011	0.425 *	−0.159
	15a	0.011	−0.019	0.298	0.387 *	−0.108	−0.308	0.407 *	0.301
September	3a	0.388 *	−0.275	0.226	−0.032	−0.517 **	−0.004	0.030	0.212
	8a	0.335	−0.235	0.187	−0.050	−0.486 **	−0.033	0.027	0.171
	15a	0.259	−0.108	0.226	0.011	−0.448 *	−0.002	0.145	0.210
Observation period	3a	0.219 **	−0.082	0.011	−0.106	−0.437 **	−0.566 **	0.298 **	0.012
	8a	0.168 *	−0.069	−0.113	−0.232 **	−0.366 **	−0.481 **	0.313 **	−0.118
	15a	−0.006	−0.142	−0.386 **	−0.515 **	−0.211 **	−0.367 **	0.288 **	−0.381 **

Note: * *p* < 0.05, ** *p* < 0.01.

## Data Availability

Data will be made available upon request.

## Appendix A

**Table A1:** Fold–Specific R^2^ Values of RF and MLR Models under 5–Fold Forward–Chaining Time–Series Cross–Validation (Model A, Eight Environmental Factors).

Stand Age/a	Model	Fold 1	Fold 2	Fold 3	Fold 4	Fold 5	Mean ± SD
3a	RF	−1.357	−0.581	−0.976	0.103	−4.045	−1.371 ± 1.422
	MLR	−0.816	−1.073	−0.704	−0.073	−1.444	−0.822 ± 0.453
8a	RF	−0.063	−2.057	−1.118	−0.169	−0.561	−0.794 ± 0.732
	MLR	−3.174	−12.085	−0.920	−0.256	−1.072	−3.501 ± 4.402
15a	RF	−6.342	−0.875	−0.677	−0.569	−4.350	−2.563 ± 2.361
	MLR	−5.957	−15.563	−2.423	−0.596	−15.173	−7.942 ± 6.304

Note: Folds 1–5 are arranged chronologically, with the training window expanding from fold to fold. Differences in R^2^ among folds indicate that temporal extrapolation performance depends on the validation period. The mean CV–R^2^ values of Model B were −0.812, −0.872, and −1.686 for 3a, 8a, and 15a, respectively.

**Table A2:** List of Abbreviations (New).

Abbreviation	Full Term	Abbreviation	Full Term
Rs	Solar radiation	MLR	Multiple linear regression
Ta	Air temperature	SHAP	SHapley Additive exPlanations
Rh	Air humidity	OOB	Out–of–bag
Ts	Soil temperature	CV	Cross–validation
Sh	soil volumetric moisture content	THB	Tissue Heat Balance
Ws	Wind speed	DOY	Day of year
Ra	Precipitation	LOESS	Locally weighted scatterplot smoothing
VPD	Vapor pressure deficit	PDP	Partial dependence plot
Q	Sap flow rate	KGE	Kling–Gupta efficiency
RF	Random forest	NSE	Nash–Sutcliffe efficiency
